# A Case of Spontaneous Ureteral Rupture Mimicking Renal Colic

**DOI:** 10.7759/cureus.35223

**Published:** 2023-02-20

**Authors:** Wilson Chiu, Muhammad Durrani, Samaresh Dasgupta, Marsha Wainwright Edwards, Carla Dugas

**Affiliations:** 1 Emergency Medicine, Inspira Medical Center, Vineland, USA

**Keywords:** double-j stent, acute renal colic, spontaneous ureteral rupture, radiology medical education, ct (computed tomography) imaging

## Abstract

A 67-year-old female presented to the emergency department with acute-onset severe left flank pain as well as nausea and vomiting. Physical examination was notable for left-sided abdominal, flank tenderness, and costovertebral angle tenderness. Laboratory workup revealed an elevated lactate of 9.2 mmol/L and elevated serum creatinine of 1.14 mg/dL, with an estimated glomerular filtration rate of 53 mL/minute/1.73m^2^. Urinalysis showed moderate leukocyte esterase with microscopy showing 12 white blood cells and three red blood cells per high-power field. CT of the abdomen and pelvis with intravenous contrast was notable for moderate amounts of left-sided perinephric and periureteric fluid without the presence of an obstructing calculus. Due to the amount of perinephric and periureteric fluid without associated nephrolithiasis, the differential diagnosis was broadened to include spontaneous ureter rupture as well as concern for malignancy. A delayed post-contrast CT scan of the abdomen and pelvis was obtained, which confirmed a spontaneous proximal and mid-ureter rupture. Spontaneous ureter rupture is a rare disease process with significant morbidity and mortality. It often poses a diagnostic dilemma due to a lack of clinical awareness and varied presentation. Diagnosis rests upon obtaining delayed post-contrast CT of the abdomen and pelvis. Currently, there are no standardized treatment guidelines, although most experts utilize minimally invasive endourological approaches in their treatment plans.

## Introduction

Spontaneous ureteral rupture, a rare and emergent urological entity, is associated with increased intraluminal pressures resulting in urinary extravasation from the ureter secondary to a non-traumatic etiology [1-4]. It is most commonly caused by ureteral calculi, but spontaneous ureteral rupture has been associated with malignancy, pregnant states, lymphoid hyperplasia, renal cysts, fibrosis, radiation, as well as different causes of intrinsic and extrinsic genitourinary system compression [5-7]. However, there have been a small number of cases where no cause was evident. Rupture may occur anywhere along the course of the ureter but is most frequently seen at the fornix or the upper ureter [8]. The true incidence of spontaneous ureteral rupture is unknown as there are only a small number of cases reported in the literature. Studies have noted that there is no sex predominance with an average reported age of 40 years [8]. The underlying pathophysiology of this disease process is not well understood at this time. Spontaneous ureteral rupture often poses a diagnostic problem due to its non-specific presentation and lack of characteristic clinical signs. Patients may be asymptomatic or present with sudden-onset flank and abdominal pain, nausea, vomiting, dysuria, and/or hematuria [1-8]. Currently, the use of delayed acquisition contrast-enhanced CT is recommended as the initial imaging modality of choice for confirming this diagnosis [1-8]. Additionally, the use of cystoscopy and retrograde pyelogram is often utilized as a part of the confirmatory process prior to urologic interventions. There are no standardized guidelines on the treatment of spontaneous ureteral rupture. Treatment is individualized and may require ureteral stent placement, percutaneous nephrostomy, endourological approaches, as well as open, laparoscopic, and robotic surgical repair [1-8]. Early identification and treatment are essential to avoid known complications such as urinoma, abscess formation, sepsis, and renal failure. This case report serves to add to the body of literature on spontaneous ureter rupture.

## Case presentation

**Figure 1:**
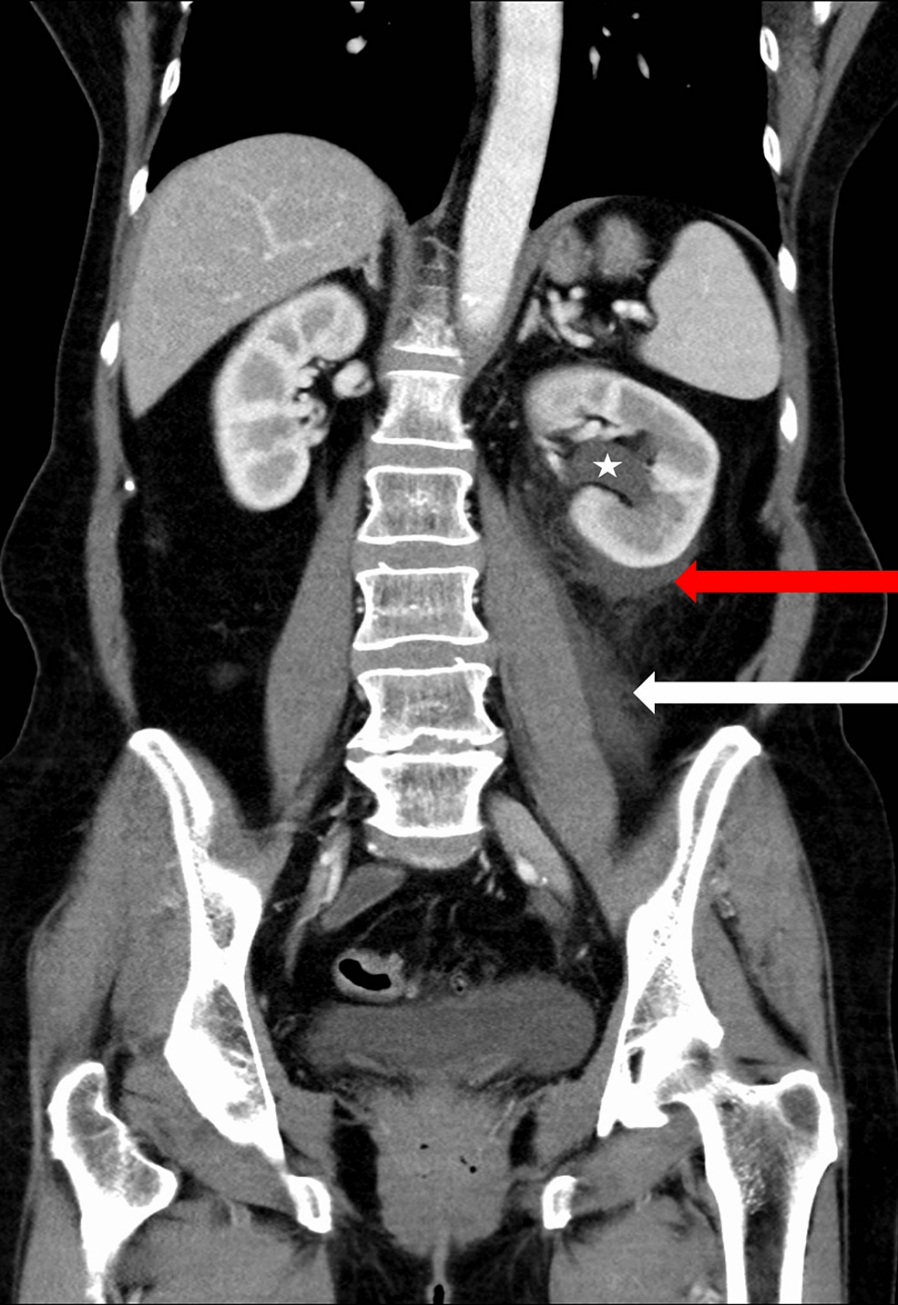
Edematous left kidney with dilation of the left renal collecting system (white arrow) as well as a moderate amount of perinephric (red arrow) and periureteric fluid (white arrow).

A 67-year-old female with a past medical history of hypertension was brought in by emergency medical services to the emergency department with an acute onset of severe left flank pain. The patient noted that the pain started several hours prior in the setting of no acute inciting factors, traumatic injuries, or changes in her activities of daily living. The pain was not alleviated or exacerbated by anything and was described as a stabbing sensation with radiation from the left flank to the left thoracic paravertebral musculature. The patient additionally endorsed associated nausea and multiple episodes of non-bloody, non-bilious emesis. The patient’s vital signs were noted to be normal, and examination revealed left lower quadrant abdominal tenderness as well as left costovertebral angle tenderness to palpation. Laboratory workup included a complete blood count, compressive metabolic panel, coagulation profile, urinalysis, and serum lactic acid testing, which were found to be unexceptional except for an elevated lactate of 9.2 mmol/L as well as an elevated serum creatinine of 1.14 mg/dL, with an estimated glomerular filtration rate of 53 mL/minute/1.73m^2^. Urinalysis demonstrated moderate leukocyte esterase with microscopy showing 12 white blood cells and three red blood cells per high-power field. The patient underwent a CT scan of the abdomen and pelvis with intravenous contrast. Imaging revealed an edematous left kidney with dilation of the left collecting system as well as a moderate amount of perinephric and periureteric fluid without the presence of an obstructing calculus (Figure 1).

**Figure 2:**
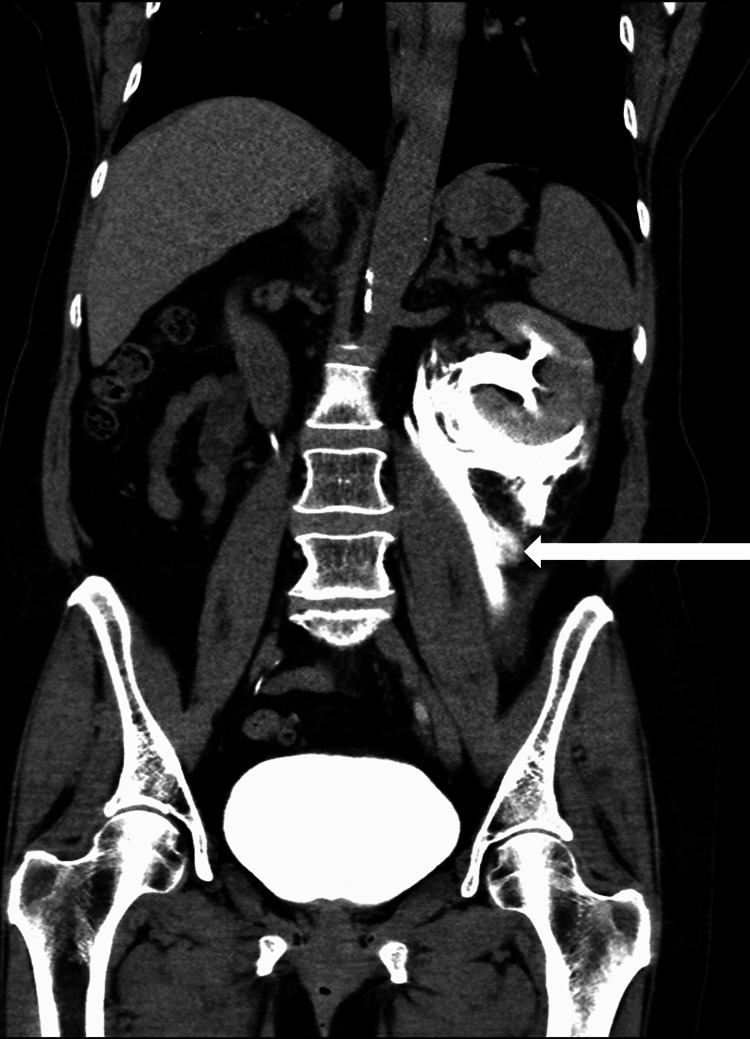
Delayed post-contrast CT scan revealing contrast extravasation from the left ureter (white arrow).

Due to the amount of perinephric and periureteric fluid present, the decision was made to obtain delayed CT imaging to exclude ureteral injury. CT with delayed images obtained after contrast injection revealed extravasation of contrast from the left proximal as well as mid-ureter (Figure 2).

The patient’s presentation was consistent with spontaneous ureter rupture. The patient received intravenous antibiotics as well as intravenous fluids and underwent cystoscopy and left-sided retrograde pyelogram. This confirmed extravasation of contrast 2 cm distal to the left ureteropelvic junction without the presence of any stones, filling defects, or other etiologies of the ureter rupture. The patient then underwent placement of a double-J stent with Foley catheter placement. The patient was discharged with oral antibiotics and outpatient follow-up for repeat cystoscopy and evaluation for possible stent removal in one month. The patient’s postoperative course was complicated by transient hematuria with outpatient CT urogram showing satisfactory positioning of the left ureteral stent without hydronephrosis, no contrast extravasation, and symmetric excretion of contrast material bilaterally. The patient ultimately underwent a repeat cystoscopy with left ureteroscopy and double-JJ stent removal with no noted stones, tumors within the ureter or within the renal pelvis, and no evidence of extravasation of the ureter.

## Discussion

Spontaneous ureteral rupture, with its varied and non-specific presentation, represents a rare but vitally important disease process to promptly identify and treat. While the majority of literature on spontaneous ureter rupture stems from the field of urology, it is a disease entity that can be encountered by many different medical specialties. Although rare, it is important to be aware of this entity as the imaging modalities to diagnose this condition are uniquely different than those used to diagnose the more common and less emergent disease processes that spontaneous ureter rupture mimics. Diagnosis relies upon obtaining delayed CT imaging of the abdomen and pelvis post-intravenous contrast. A delayed post-contrast CT scan is not a commonly ordered imaging study. When this is coupled with a general lack of clinical awareness of spontaneous ureter rupture as well, it is easy to see why spontaneous ureteral rupture often poses a diagnostic problem. Our patient’s initial presentation of severe flank pain mimicked renal colic and our workup was tailored to ruling out nephrolithiasis. The appearance of perinephric fluid and periureteric fluid on the initial contrast-enhanced CT scan is what altered us to the possibility of a potential ureter rupture. Once this disease entity was entertained, we were able to obtain a delayed CT scan post-intravenous contrast to confirm the diagnosis. In this particular case, the repeat CT scan was performed close to two hours following the initial contrast injection, but studies have shown that delayed images obtained as early as 5-20 minutes after contrast injection can confirm this diagnosis [5,9]. The use of delayed imaging can also additionally differentiate a perinephric abscess as well as a forniceal rupture from a ureter rupture [8]. Treatment of spontaneous ureter rupture is an emerging field of study as there are no current standardized guidelines. Many studies have noted the benefits of minimally invasive endourological approaches such as double-J stent and percutaneous drainage to achieve unobstructed urinary outflow and healing of the perforation [10]. We hope to increase clinical awareness of this rare disease entity as well as to add to the body of literature on spontaneous ureter rupture.

## Conclusions

Spontaneous ureter rupture is a rare and emergent disease process that may often be misdiagnosed due to a lack of clinical awareness. It is associated with significant rates of morbidity and mortality. Delay in diagnosis and treatment may lead to complications such as renal failure, abscess formation, urinoma, as well as septic shock. Prompt recognition and awareness of this disease state are essential to mitigate the complications and mortality associated with it. This case report should serve to reinforce this rare disease process as well as to reinforce the unique diagnostic modality necessary to confirm the diagnosis.
